# Ultrasonographic evaluation of placental cord insertion at different gestational ages in low-risk singleton pregnancies: a predictive algorithm

**Published:** 2016-03-28

**Authors:** F Padula, AS Laganà, SG Vitale, L Mangiafico, L D’Emidio, P Cignini, M Giorlandino, FA Gulino, S Capriglione, C Giorlandino

**Affiliations:** Department of Prenatal Diagnosis. Altamedica, Fetal-Maternal Medical Center, 00198 Rome (Italy); Unit of Gynecology and Obstetrics, Department of Human Pathology in Adulthood and Childhood “G. Barresi”. University of Messina, 98125 Messina (Italy).; Gynaecology and Obstetrics Section. Department of Medical Surgical Specialties. University of Catania, 95100 Catania (Italy).; Department of Obstetrics and Gynaecology. Campus Bio Medico University of Rome, 00128 Rome (Italy).

**Keywords:** Accuracy, fetal ultrasound, ultrasonography, placental cord insertion, predictive model, fetal ultrasound, umbilical cord

## Abstract

**Objective:**

To evaluate the accuracy of ultrasound in visualizing placental cord insertion (PCI) at different gestational ages in order to recommend the most feasible period during pregnancy to identify it. Secondary aim was to propose a predictive algorithm for PCI visualization.

**Methods:**

We performed a single-center, prospective cohort study. We enrolled patients with singleton low-risk pregnancies who underwent fetal ultrasound scan at different gestational ages. We excluded patients with body mass index of 30 Kg/m2 or more, uterine fibroids larger than 5 cm, high-risk pregnancies, fetal weight lower than < 10° percentile or higher than > 90° percentile, increased (“deep pocket” > 80 mm) or decreased (“deep pocket” < 20 mm) amniotic fluid.

**Results:**

Among the 468 recruited patients, the visualization of PCI was not possible in 5.77% of the cases. Furthermore, we showed that PCI visualization was lower as the gestational age increased (p = 0.049) and more difficult in case of posterior placenta (p = 0.001).

**Conclusions:**

PCI should be evaluated in the first trimester or as early as possible during the second trimester. Moreover, we propose a feasible model to predict the possibility of PCI visualization according to gestational age and uterine site of implantation.

## Introduction

Accumulating evidence suggests that abnormal cord insertion (ACI) seems to be associated with impaired development and function of the placenta, playing a detrimental role for fetal growth (Misra et al., 2009). Furthermore, velamentous and marginal insertions of the umbilical cord are associated with an increased risk of adverse outcomes such as placenta praevia and placental abruption (Räisänen et al., 2012). In addition, in case of ACI the risk of pre-eclampsia, preterm birth and delivery by acute caesarean are doubled, as is the risk of low Apgar score, transfer to a Neonatal Intensive Care Unit (NICU), low birthweight and congenital malformations (Uyanwah-Akpom and Fox, 1977). Velamentous and marginal insertions are reported to occur in 0.5-2.4% and 8.5% of all pregnancies, respectively (Robinson et al., 1983), with the prevalence being higher in multiple pregnancies (Kent et al., 2011) and in pregnancies conceived with the aid of assisted reproductive technology (Delbaere et al., 2007). Apart from twin gestation and assisted reproductive technology, a large population-based study identified bleeding in pregnancy, advanced maternal age, maternal chronic disease, female fetus and previous pregnancy with velamentous and marginal cord insertions as other risk factors for ACI (Ebbing et al., 2013). To date, placental cord insertion (PCI) can be reliably detected prenatally by gray-scale and color Doppler ultrasound (Gizzo et al., 2015). Conversely, 3D ultrasound seems to perform poorly at evaluating PCI, being less efficient due to poorquality resolution and far more time-consuming than the combined use of gray-scale and color Doppler ultrasound (Sepulveda et al., 2003). According to Nomiyama et al. (1998), at 18-20 gestational weeks the sonographic identification of ACI has a sensitivity of 100%, a specificity of 99.8%, a positive predictive value of 83% and a negative predictive value of 100%. Nevertheless, others suggested to check for ACI at the time of the 11- to 14-week scan, since this allows close surveillance of the pregnancy for potential complications associated with this condition (Sepulveda, 2006). Considering all these data, the aim of our work was to evaluate the accuracy of ultrasound in visualizing PCI at different gestational ages in order to recommend the most feasible period during pregnancy to identify it. Secondary aim was to propose a predictive algorithm for PCI visualization at different gestational ages.

## Methods

We performed a single-center, prospective cohort study. Our cohort consisted of consecutive patients with singleton low-risk pregnancies, who underwent fetal ultrasound scan at different gestational age from the second trimester, in Altamedica Medical Center (Rome, Italy), between July and September 2014. The study was designed in accordance with the Helsinki Declaration, conforms the Committee on Publication Ethics (COPE) guidelines (http://publicationethics.org/) and was approved by the Institutional Review Board (IRB) of the Clinic in which it was performed. As standard protocol of the clinic in which the study was performed, each patient was informed and signed an informed consent allowing data collection for research purposes. All the design, analysis, interpretation of data, drafting and revisions followed the “Strengthening the Reporting of Observational Studies in Epidemiology” (STROBE) Statement: guidelines for reporting observational studies (von Elm et al., 2008), available through the EQUATOR (Enhancing the QUAlity and Transparency Of health Research) network (http://www.equator-network.org/).

We excluded patients with body mass index of 30  Kg/m2 or more, patients with uterine fibroids larger than 5 cm, high-risk pregnancies (including maternal diseases such as hypertension or diabetes), estimated fetal weight lower than < 10° percentile or higher than > 90° percentile, increased (“deep pocket” > 80 mm) or decreased (“deep pocket” < 20 mm) amniotic fluid. PCI evaluation was performed in all cases by different gynecologists with at least 9 years of experience in fetal ultrasound and about 1000 scans/year, using a Samsung Accuvix A30, General Electrics Voluson 730 Pro or Voluson E8 with a 2D (4.5-16.5 MHz) transabdominal probe. In case of difficult visualization of PCI, a 2D (5-9 MHz) transvaginal probe was used. Placental localization was evaluated through longitudinal and transverse scans and classified as fundal, anterior and posterior. Moreover, the fetal and placental sites of insertion, the number of vessels, and eventually the presence of knots, cysts and tumors were recorded. Cord insertion at the placental site was systematically assessed through color Doppler analysis to assure that the cord vessels continue into the placenta, and classified as central/paracentral, marginal (if the umbilical cord inserted within 2 cm of the placental edge) and velamentous (if the umbilical cord inserts into the chorio-amniotic membranes, outside the placental margin). We further evaluated the number of vessels within the umbilical cord and, if the evaluation was uncertain, color Doppler visualization of the fetal intraabdominal vessels running bilaterally along the bladder was performed to confirm the presence of two umbilical arteries (Geipel et al., 2000).

MedCalc Software 12.4.0.0 was used to analyze the correlation between ultrasound features (placental localization, type of umbilical cord insertion and gestational age) and PCI visualization by Spearman’s test. Accuracy, sensitivity, specificity, positive and negative predictive value (PPV and NPV) were calculated. The level of statistical significance was set at p < 0.05. In the second part of the study, basing on the estimation of receiver operating characteristic (ROC) curve, stepwise logistic regression was performed in order to identify a predictive algorithm for PCI visualization by ultrasound.

## Results

**Fig. 1:**
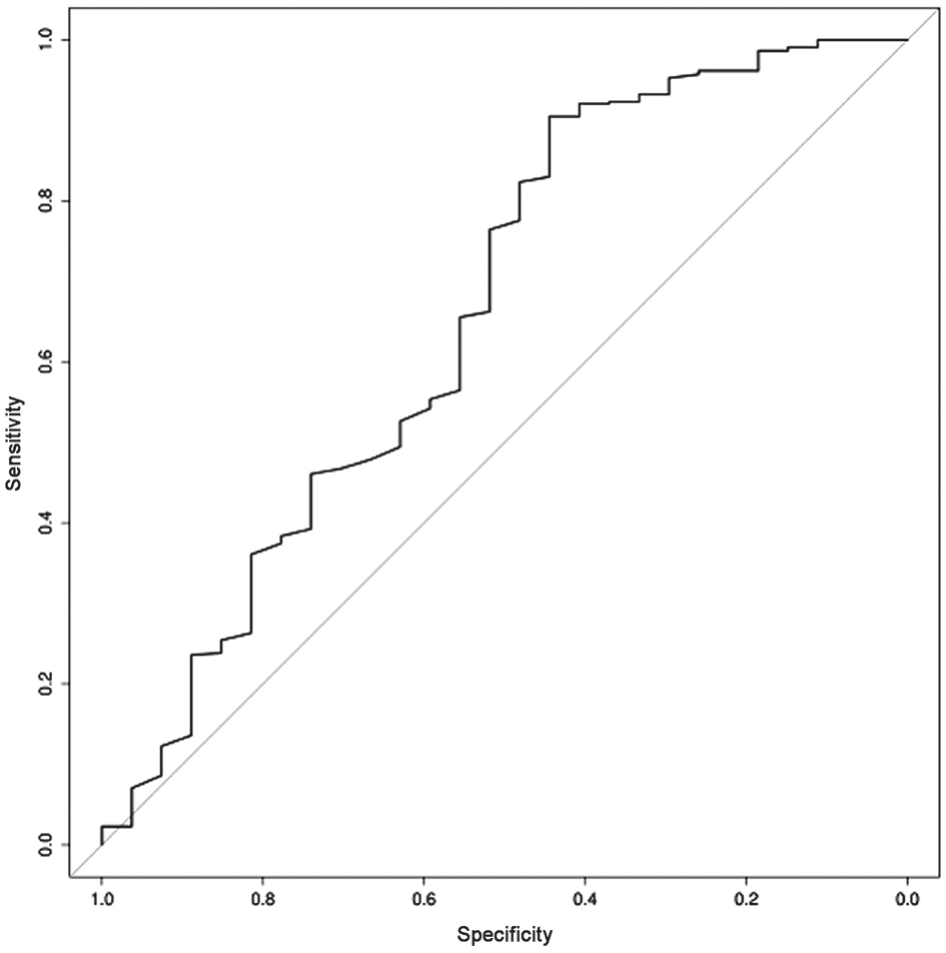
— Receiver operating characteristic (ROC) curve of predictive algorithm for PCI visualization by ultrasound.

We enrolled 468 patients, fitting out inclusion/exclusion criteria. The mean age of enrolled patients was 36.5 ± 12.4 (range 18-48) years; mean gestational age was 21.83 ± 7.1 (range 16-38) weeks and mean BMI was 23.1 ± 6.4 (range 18-29) Kg/m2. Table I summarizes the percentages of PCI visualization at different gestational ages. Table II summarizes the percentages of PCI visualization and definition according to uterine site of implantation. In our cohort, the visualization of PCI was not possible in 5.77% of the cases. Furthermore, we showed that PCI visualization was lower as the gestational age increased (p = 0.049) and more difficult in case of posterior placenta (p = 0.001). In the second part of the study, on the basis of these statistical evaluations, the significant variables were included into a multivariate logistic regression model in order to identify the statistically relevance of each parameter in the predictive model. Hosmer-Lemeshow’s test was used to verify the overall model significance and its goodness of fit (p < 0.0001). Therefore, we obtained the equation of logistic regression to determine the probability to obtain umbilical cord visualization at different gestational ages:

$P=1/1+e^{\left(-4.62999+0.09245*X_{1}-0.85659*X_{2}\right)}$

where, X1 = gestational age (expressed in weeks) and X2 = anterior placenta (yes = 1 or no = 0. Applying this formula in our cohort of patients, we obtained an accuracy of 65.9% in terms of sensitivity and specificity (95% CI 0.491-0.732), as demonstrated by the ROC curve in Figure 1.

**Table I:** — Percentages of placental cord insertion (PCI) visualization at different gestational ages.

weeks	N	N of	%
		visualization	
16-19	184	174	94.6
20-23	180	177	98.3
24-29	23	21	91.3
30-34	63	56	88.9
35-38	18	13	72.2

**Table II:** — Percentages of placental cord insertion (PCI) visualization and definition according to uterine site of implantation.

Placental localization	Placental cord insertion					
	Central	Paracentral	Marginal	Velamentous	Unknown	Total
Anterior	177 (77.6%)	29 (12.7%)	12 (5.3%)	2 (0.9%)	8 (3.5%)	228
Posterior	166 (71.6%)	28 (12.1%)	20 (8.6%)	-	18 (7.8%)	232
Fundal	6 (75%)	1 (12.5%)	-	-	1 (12.5%)	8
Total	349 (74.6%)	58 (12.4%)	32 (6.8%)	2 (0.4%)	27 (5.8%)	468

## Discussion

Intrapartum and postpartum events could depend, at least in part, by maternal condition (Ciancimino et al., 2014; Corrado et al., 2014; Laganà et al., 2015b) as well as by obstetric techniques (Burgio et al., 2015; Laganà et al., 2015a; Muscatello et al., 2015), used during labour and delivery. Indeed, to date prenatal identification of risk conditions plays a pivotal role in obstetrics. In this view, the fast improvement of ultrasound techniques already changed the scenario of pregnancy management. As far as the topic of our work is concerned, accumulating evidence suggests that placentas with a non-central cord insertion have a sparser chorionic vascular distribution, as measured by the relative vascular distance (Yampolsky et al., 2009). Thus, even if typically a placenta with a non-central insertion is of a normal round shape, its vasculature is less metabolically effective. According to the recent work by Pathak et al. (2010), cord coiling, umbilical cord insertion and placental shape variables are not associated with higher incidence of pre-eclampsia, pregnancy-induced hypertension, gestational diabetes mellitus and intrauterine growth restriction. Conversely, it is widely accepted that velamentous and marginal cord insertions (ACI) are unmistakably associated with worse obstetric outcomes (Uyanwah-Akpom and Fox 1977; Räisänen et al., 2012; Ebbing et al., 2013). In this regard, the prenatal detection of these clear pathologic conditions can enormously improve the level of attention during pregnancy monitoring, although the International Society of Ultrasound in Obstetrics and Gynecology (ISUOG)’s practice guidelines for performance of the routine mid-trimester fetal ultrasound scan recommend the visualization of the PCI only in multiple pregnancies (Salomon et al., 2011). For singleton pregnancies, the recommended minimum requirements for placenta characterization are: position, presence/ absence of masses and accessory lobe. As already evidenced, targeted sonographic examination of the placental site of umbilical cord insertion will reveal abnormal PCI, although distinguishing the specific type of abnormal insertion may require the use of color Doppler (Pretorius et al., 1996; Di Salvo et al., 1998; Nomiyama et al., 1998). Considering that the risks associated with ACI were already well established for twin gestation (Malinowski, 2003; Ebbing et al., 2013), our work focused on singleton pregnancies. In full agreement with previous literature we showed that PCI visualization was lower as the gestational age increased and more difficult in case of posterior placenta (Heinonen et al., 1996). Furthermore, as a result of our data we proposed a feasible model to predict the possibility of PCI visualization according to gestational age and uterine site of implantation. To the best of our knowledge, this is the first study which proposes this kind of predictive model.

Nevertheless, several limitations may have affected our analysis: due to the restrictive exclusion criteria (BMI of 30 Kg/m2 or more, growth restricted foetuses, pregnancies with oligohydramnios and polyhydramnios), we excluded a priori about 15% of all pregnant women already at the start of this study, together with the percentage of cases in which PCI visualization was not possible (5.7%), lead to a possible application of the predictive algorithm in about 80% of the low-risk pregnancies. Furthermore, the ultrasound examination was always performed by expert sonographers, which does not reflect the real hospital-based clinical settings. Last but not least, we did not collect information at birth, since our aim was simply to calculate the possibility to visualize the PCI during pregnancy. According to our data analysis, PCI should be evaluated in the first trimester or in any case as early as possible during the second trimester. PCI visualization was not possible in a considerable percentage of the cases even in ideal patients and when the scan is performed by expert sonographers, as in our setting. This concept is in line with another study where we underline that there is always a small percentage of incomplete foetal anatomic surveys during the second-trimester scan, which cannot be modified (Padula et al., 2015). For this reason, patients should be informed about this limit and invited to come back for a second-look if the scan was not exhaustive enough.

According to our data there is a need for further studies on larger cohorts and with greater statistical power which may confirm the accuracy of the proposed predictive algorithm. Furthermore, we strongly solicit future studies which would apply the proposed algorithm to the classes of patients that we excluded from the current analysis.
